# Inequalities in infant mortality in Brazil at subnational levels in Brazil, 1990 to 2015

**DOI:** 10.1186/s12963-020-00208-1

**Published:** 2020-09-30

**Authors:** Célia Landmann Szwarcwald, Wanessa da Silva de Almeida, Renato Azeredo Teixeira, Elisabeth Barboza França, Marina Jorge de Miranda, Deborah Carvalho Malta

**Affiliations:** 1 Institute of Communication and Scientific and Technological Information in Health, Oswaldo Cruz Foundation, Rio de Janeiro, Brazil; 2 Faculty of Medicine, Federal University of Minas Gerais, Belo Horizonte, Brazil; 3 Secretariat of Health Surveillance, Ministry of Health, Brasília, Brazil; 4 School of Nursing, Federal University of Minas Gerais, Belo Horizonte, Brazil

**Keywords:** Infant mortality rate, GBD, Geographic inequalities, Income, Brazil

## Abstract

**Background:**

In this study, infant mortality rate (IMR) inequalities are analyzed from 1990 to 2015 in different geographic scales.

**Methods:**

The Ministry of Health (MoH) IMR estimates by Federative Units (FU) were compared to those obtained by the Global Burden of Disease (GBD) group. In order to measure the inequalities of the IMR by FU, the ratios from highest to lowest from 1990 to 2015 were calculated. Maps were elaborated in 2000, 2010, and 2015 at the municipality level. To analyze the effect of income, IMR inequalities by GDP per capita were analyzed, comparing Brazil and the FU to other same-income level countries in 2015, and the IMR municipal estimates were analyzed by income deciles, in 2000 and 2010.

**Results:**

IMR decreased from 47.1 to 13.4 per 1000 live births (LB) from 1990 to 2015, with an annual decrease rate of 4.9%. The decline was less pronounced for the early neonatal annual rate (3.5%). The Northeast region showed the most significant annual decline (6.2%). The IMR estimates carried out by the GBD were about 20% higher than those obtained by the MoH, but in terms of their inequalities, the ratio from the highest to the lowest IMR among the 27 FU decreased from 4 to 2, for both methods. The percentage of municipalities with IMR higher than 40 per 1000 LB decreased from 23% to 2%, between 2000 and 2015. Comparing the IMR distribution by income deciles, all inequality measures of the IMR decreased markedly from 2000 to 2010.

**Conclusion:**

The results showed a marked decrease in the IMR inequalities in Brazil, regardless of the geographic breakdown and the calculation method. Despite clear signs of progress in curbing infant mortality, there are still challenges in reducing its level, such as the concentration of deaths in the early neonatal period, and the specific increases of post neonatal mortality in 2016, after the recent cuts in social investments.

## Background

In the last 30 years, Brazil underwent several social transformations. Increased access to urban infrastructure, improved female education, and increased female labor market participation have resulted in a sharp drop in fertility rates [1, 2]. In the 2000s, government income transfer programs, such as the “Bolsa Família”, led to changes in income distribution and reduction of health inequalities [3].

The country has moved from a multiple system to a unified health system, with profound changes to public policies [4]. A set of programs was formulated by the Ministry of Health (MoH) focused on reducing infant mortality. These included considerable expansion of primary health care coverage through the Family Health Strategy [5] and the universalization of access to immunization [6]. During those years, the municipalities developed actions to make the implementation of these programs feasible at the local level, with organization criteria defined by the federal government and others bodies of municipal management.

International agreements such as the achievement of the millennium goals and the recent agreement on the Sustainable Development Goals (Agenda 2030) have raised the challenge of broadly improving maternal and child health conditions [7, 8], promoting the need to evaluate the programs and the construction of health indicators that could assess the achievement of goals [9, 10].

Despite the availability of the Mortality Information System (SIM) from 1976 and the Live Birth Information System (SINASC) from1994, the inadequate registry and the lack of information quality, especially in the Federative Units located in the North and Northeast regions, limited the use of the registration systems at the national level [11]. Until 1990, the indirect demographic methods based on household surveys were used to estimate the probabilities of death by age group, specifically in the first year of life [2]. However, the inaccuracies found in the mortality estimates based on surveys hampered the monitoring of progress gains [12, 13].

Having recognized the importance of monitoring vital information, many government initiatives were adopted to increase the completeness and improve the quality of information, such as the technological improvement of the MoH information systems, and goals were established to increase the completeness of death information in states and municipalities and the expansion of surveillance actions to reduce maternal, fetal, and child deaths [14]. Projects were developed to detect non-notified vital events. Some studies of pro-active search of vital events were conducted in selected municipalities, investigating possible sources of information to find deaths not informed to the MoH [15].

Parallel to the improvement of information gathering, based on the results of the active searches of deaths and births, in 2000 a method was adopted to correct vital information which allowed the use of continuous registry data for the elaboration of mother–child health indicators in different geographic scales [16, 17].

This paper analyzes the inequalities of infant mortality in Brazil from 1990 to 2015, estimated at subnational levels, such as geographic macro-regions, Federative Units (FU), and municipalities. The estimates by FU were compared to those obtained by the Global Burden of Disease (GBD) group [18]. To investigate the effects of the per capita income of the municipality and the changes in income distribution, the IMR estimates for all Brazilian municipalities were analyzed by income deciles, in 2000 and 2010, and the IMR inequalities by income were compared in these two-year censuses.

## Methods

Brazil is divided politically and geographically into five macro-regions (North, Northeast, Southeast, South, and Center-West) with distinct physical, demographic, and socioeconomic characteristics. The North and Northeast regions have the worse socioeconomic development levels. The country is subdivided into 27 Federative Units (FU), which comprise a total of 5570 municipalities, with significant variation in the population size, from 800 to 12 million inhabitants.

In this study, until the year 2000, the infant mortality indicators were estimated by a mixed model with direct estimation for eight FU and indirect estimation by demographic methods for the other FU [19]. For the subsequent years, the indicators were directly estimated by the vital information systems, using, when necessary, adjustment factors for municipalities with incomplete information. The estimates were broken down by infant mortality components: early neonatal (0–6 days), late neonatal (7–21 days), post-neonatal (28 days or more).

### Adjustment of vital information

The method adopted for the IMR estimation consists in using correction factors according to the level of completeness of death and live birth information. The application of the method at the municipality level made the use of the continuous registry data possible for the elaboration of mother–child health indicators in all Federative Units (FU) and Brazilian municipalities starting from 2000 [16, 17].

The adjustment factors were estimated from the results of the active search studies of death and births conducted in 2010 and 2014. The sampling process and the method used for correcting the vital statistics by municipality were described in detail in previous publications [16, 17].

Briefly, the method consists of characterizing the level of completeness of mortality information by means of the age-standardized general mortality rate (GMR) calculated in all Brazilian municipalities. When the GMR does not achieve the expected value in a certain municipality, correction factors are used to adjust the number of deaths, considering deaths among children aged less than one year and deaths between individuals one year of age or over separately [16, 17]. A similar procedure was used for the adjustment of the number of live births. The ratio between the informed and estimated LB [17] was used as the indicator to evaluate the level of completeness of live birth information.

### The global burden disease (GBD) study method

The Global Burden of Disease Study in 2017 estimated national and subnational infant mortality trends for each country, between 1950 and 2017 [18]. The time trend of mortality was estimated through a time-space Gaussian regression model, considering three covariates: per capita income, mean number of schooling years for women aged 15–49 years and the crude mortality rate due to infection with Human immunodeficiency virus (HIV). Different sources of vital information were used for each country, using the Vital Registry (VR) in countries with complete VR, and complete birth backgrounds in countries without complete VR.

Mortality for individuals with less than five years of age required a modeling of the death probabilities in the first five years of life (5q0), for each gender. Then, in separate models, the death probabilities were estimated for each gender and age group, specifically in the first year of life (infant mortality) and of 1–5 years of life (childhood mortality).

### Infant mortality inequalities

The IMR was estimated for the 27 FU and the five geographic macro-regions, from 1990 to 2015 to analyze geographic inequalities. The estimates among the FU and geographic macro-regions were compared to the ones obtained by the GBD. The ratio from highest to lowest estimate was calculated to measure IMR’s geographic inequalities by FU. IMR inequalities by per capita GDP were analyzed as well, by comparing Brazil and the Federative Units with other countries of the same income level in 2015 [20].

At the municipality level, IMR estimates were presented in the form of maps in the years 2000, 2010, and 2015, in order to investigate spatial inequalities.

In order to analyze the effects of the per capita income of the municipality and the changes in income distribution, the Brazilian Atlas of Human Development [21] was used as the source of information for the municipality’s per capita income, in the years 2000 and 2010. The IMR estimates for all Brazilian municipalities were analyzed by income deciles, in 2000 and 2010, and the IMR inequalities by income [22] were compared in these two-year censuses.

## Results

The completeness of vital information significantly improved over the analyzed period (Table 1). In 1995, the percentage of informed number of deaths and births was less than 85%. With the progressive increase of vital event notifications, both SINASC and SIM are now almost complete, reaching values of completeness above 96%. Regarding infant deaths, the proportion of deaths reported to SIM increased from 52.0% in 1990 to 88.5% in 2015.

**Table 1:** Mortality indicators (Brazil, 1990–2015)

Indicators	Year					
	1990	1995	2000	2005	2010	2015
SIM^a^ completeness (%)	76.6	83.6	91.0	93.2	94.2	96.7
Proportion (%) of child deaths informed to the SIM^a^	52.0	65.7	74.1	80.6	84.9	88.5
SINASC^b^ completeness (%)	-	82.4	92.5	95.5	95.9	96.4
Infant mortality rate (/1000 LB)	47.1	35.1	26.1	20.4	16.0	13.5
Post-neonatal mortality rate (/1000 LB)	24.0	15.2	9.4	6.8	4.9	4.0
Early neonatal mortality rate (/1000 LB)	17.7	15.7	13.1	10.5	8.1	7.3
Late neonatal mortality rate (/1000 LB)	5.4	4.2	3.6	3.1	3.0	2.2

^a^Mortality Information System

^b^Live Birth Information System

The results of Table 1 also show a sharp decline in mortality in the first year of life, which decreased from 47.1 to 13.5 per 1000 LB between 1990 and 2015, with a rate of decrease of 4.9% per year for the whole country.

The analysis by component of infant mortality, however, shows a more pronounced decrease in post-neonatal mortality between 1990 and 2015, from 24.0 to 4.0 per 1000 LB, than in the neonatal component, which decreased from 23.1 to 9.5 per 1000 LB, with high concentration of deaths in the early neonatal period. In 2015, 70% of infant deaths occurred in the neonatal period, 54% in the first week of life (Table 1).

Table 2 shows the IMR estimates by FU and geographic macro-regions. Between 1990 and 2015, the Northeast region showed the most significant annual reduction rate at 6.2%, while other regions averaged 4% approximately, contributing to a decrease in the regional inequality in infant mortality throughout the period. The ratio of inequality, estimated by the ratio of the highest to the lowest estimate of IMR in the set of 27 FU, decreased from 3.9 in 1990 to about 2 from 2005.

**Table 2:** IMR estimates by Federative Units and geographic macro-region (Brazil, 1990–2015)

Region and FU	1990		1995		2000		2005		2010		2015		Annual decrease rate	
	MoH	GBD	MoH	GBD	MoH	GBD	MoH	GBD	MoH	GBD	MoH	GBD	MoH	GBD
**North region**	**45.9**	**55.2**	**38.8**	**43.4**	**32.8**	**34.9**	**27.1**	**25.1**	**21.0**	**21.1**	**17.5**	**18.0**	**3.8**	**4.4**
Rondônia	42.6	53.7	36.9	30.5	31.9	31.6	24.9	20.3	18.9	18.6	14.7	17.8	4.2	4.3
Acre	56.5	74.2	40.6	60.1	29.2	48.6	24.9	37.5	20.4	29.5	17.6	22.9	4.6	4.6
Amazonas	44.5	47.8	39.4	41.1	34.8	33.0	25.7	19.6	20.6	18.5	18.2	19.4	3.5	3.5
Roraima	39.7	42.3	29.7	35.6	22.2	26.7	22.3	20.4	18.0	17.4	19.1	18.6	2.9	3.2
Pará	46.2	57.2	38.6	45.4	32.3	35.1	28.6	27.0	21.5	21.8	17.6	16.8	3.8	4.8
Amapá	38.1	41.5	35.4	40.6	32.9	34.0	27.8	22.2	25.4	22.8	20.8	23.5	2.4	2.2
Tocantins	44.9	61.3	40.7	46.9	36.9	37.8	28.2	28.9	20.5	22.3	14.2	16.0	4.5	5.2
**Northeast region**	**75.8**	**85.1**	**52.2**	**62.8**	**35.9**	**47.1**	**25.9**	**34.6**	**19.1**	**25.4**	**15.1**	**18.1**	**6.2**	**6.0**
Maranhão	76.6	88.2	53.1	62.0	36.8	46.8	26.5	34.5	21.9	25.3	16.5	17.3	6.0	6.3
Piauí	65.0	65.1	49.6	52.5	37.8	42.3	27.3	33.6	20.7	24.3	16.3	16.9	5.4	5.3
Ceará	79.5	99.8	54.1	73.0	36.8	52.6	24.2	35.7	16.2	24.6	12.7	16.6	7.1	6.9
Rio Grande do Norte	75.7	74.1	51.1	57.3	34.5	39.6	25.3	26.5	17.2	19.3	14.2	14.6	6.5	6.3
Paraíba	81.9	65.2	56.6	45.8	39.2	32.6	25.3	24.7	18.2	17.6	13.0	12.1	7.1	6.5
Pernambuco	77.0	90.4	51.1	64.8	34.0	48.4	23.4	34.5	17.0	25.3	13.4	18.2	6.8	6.2
Alagoas	102.2	111.0	62.1	75.7	37.7	56.8	27.8	38.2	18.6	26.5	14.5	18.6	7.5	6.9
Sergipe	65.6	66.0	49.7	54.3	37.7	42.3	27.2	32.3	18.2	24.6	15.4	18.6	5.6	4.9
Bahia	66.0	80.2	47.8	62.1	34.6	48.3	27.5	38.2	21.0	29.8	17.5	22.2	5.2	5.0
**Southeast region**	**32.6**	**38.0**	**25.6**	**30.6**	**20.1**	**24.1**	**16.0**	**17.6**	**13.4**	**15.8**	**12.2**	**14.5**	**3.9**	**3.8**
Minas Gerais	39.0	39.4	31.7	32.4	25.7	29.5	20.3	24.9	16.2	19.2	13.7	14.6	4.1	3.9
Espírito Santo	33.2	39.0	24.5	29.9	18.1	22.9	15.4	21.1	11.9	17.1	11.7	15.0	4.1	3.7
Rio de Janeiro	32.3	33.0	25.8	25.9	20.5	20.1	16.8	15.1	14.3	15.0	13.1	15.0	3.5	3.1
São Paulo	30.8	39.1	23.1	31.7	17.4	23.1	13.8	14.5	12.0	14.3	11.3	14.2	3.9	4.0
**South region**	**28.3**	**34.2**	**21.9**	**29.2**	**16.9**	**23.7**	**14.1**	**19.5**	**11.6**	**16.6**	**10.7**	**15.3**	**3.8**	**3.2**
Paraná	35.1	38.9	25.8	30.6	19.0	25.0	14.7	22.4	12.0	18.5	11.1	16.3	4.5	3.4
Santa Catarina	33.6	38.4	23.1	33.1	15.9	27.1	12.9	22.2	11.2	18.0	9.9	15.1	4.8	3.7
Rio Grande do Sul	26.2	27.0	20.1	25.6	15.3	20.3	14.0	14.6	11.3	13.4	10.8	14.3	3.5	2.5
**Center-west region**	**34.4**	**38.1**	**27.7**	**33.0**	**22.3**	**24.0**	**19.3**	**18.2**	**15.9**	**15.7**	**13.2**	**15.6**	**3.8**	**3.5**
Mato Grosso do Sul	32.3	37.3	28.1	37.0	24.4	30.0	19.8	22.2	15.4	18.2	12.9	16.0	3.6	3.3
Mato Grosso	37.6	42.5	33.2	34.8	29.4	27.9	24.0	20.6	19.6	16.9	14.9	17.4	3.6	3.5
Goiás	35.1	37.5	27.3	31.4	21.2	20.2	19.1	15.5	15.9	14.4	13.4	15.5	3.8	3.5
Federal District	28.9	34.9	21.0	30.5	15.3	22.3	13.6	17.6	12.2	14.8	10.9	13.4	3.8	3.8
**Brazil**	**47.1**	**55.7**	**35.1**	**43.3**	**26.1**	**33.7**	**20.4**	**25.2**	**16.0**	**20.1**	**13.5**	**16.4**	**4.9**	**4.8**
**Ratio between the highest and lowest value**	**3.9**	**4.1**	**3.1**	**3.0**	**2.6**	**2.8**	**2.1**	**2.6**	**2.3**	**2.2**	**2.1**	**1.9**	-	-
**Correlations between the GBD and MoH estimates**	**0.951**		**0.910**		**0.820**		**0.685**		**0.648**		**0.783**		-	-

The comparison of the IMR GBD estimates with the national ones shows ever greater magnitudes for the GBD rates. As to the magnitude of the IMR GBD estimates by FU and region, the greatest differences are found in the South Region. In the year 2015, the GBD estimates for Paraná and Santa Catarina were 50% higher than the rates calculated directly through registry information. Besides, for the same year, the GBD estimates for Pernambuco and Ceará, states located in the Northeast, were more than 30% higher than those estimated by the MoH. For Brazil, the ratio between the GBD and the MoH estimates ranged from 1.18 in 1990 to 1.29 in 2000 but decreased to 1.21 in 2015. As the ratios between the rates estimated by GBD and MoH were similar in the years 1990 and 2015, the annual rates of decrease calculated by the IMR variation from 1990 to 2015 were also similar (Table 2).

Regarding the geographical inequalities, the ratio of the highest to the lowest IMR estimate by the GBD per FU had the same temporal trend as the MoH estimates, decreasing from 4.1 in 1990 to 1.9 in 2015. The correlations between GBD and MoH estimates were high and significant in all years. In the year 1990, the correlation had the highest value of 0.951, and in 2010 the lowest value of 0.648. In 2015, the correlation was 0.783 (Table 2).

**Fig. 1:**
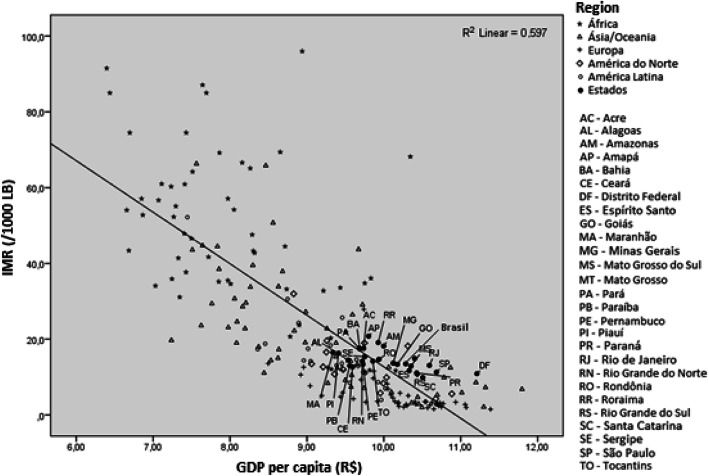
Comparison between the IMR in world countries and the IMR among FU by per capita GDP, 2015

Figure 1 shows that the current magnitude of the national rate, estimated at 13.5 per 1000 live births in the year 2015, approaches the regression line that relates IMR estimates to the per capita GDP of the various countries of the world, that is, matches the expected IMR in the countries with equivalent per capita GBD. It is interesting to note that several states in the Northeast Region, such as Rio Grande do Norte, Pernambuco, Ceará, Maranhão, Piauí, Alagoas, Paraíba, and Sergipe, are below the regression line, thus showing a lower level of IMR than expected Gross Domestic Product (GDP) per capita. On the other hand, the Federal District, São Paulo, and Rio de Janeiro, which present the highest GDP per capita values in Brazil, have higher rates than expected.

**Fig. 2:**
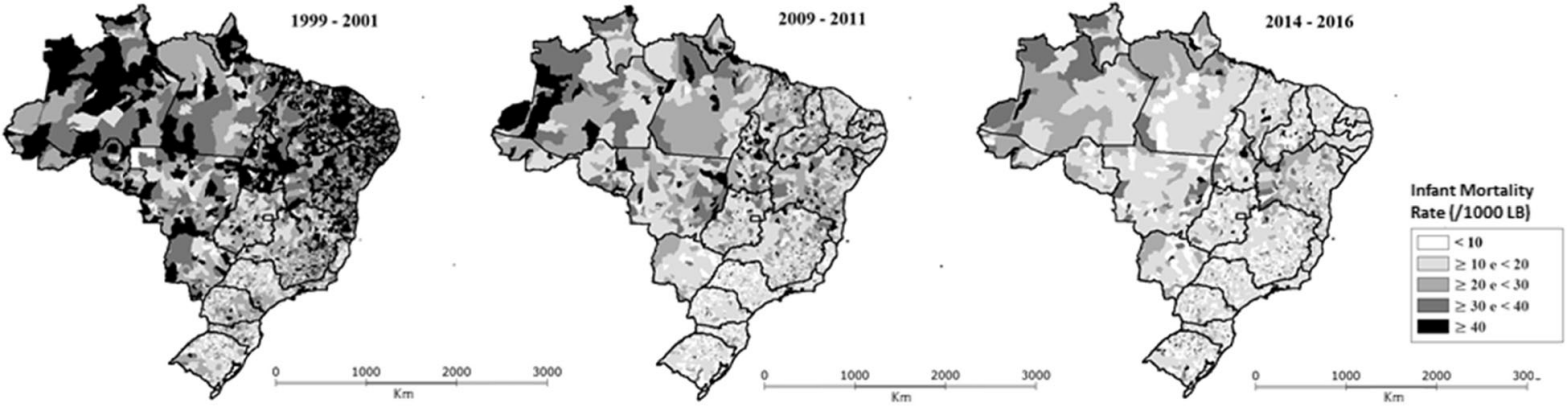
Spatio-temporal IMR trends (Brazil, 1999–2016)

Figure 2 shows the decrease in the IMR spatial inequality in Brazilian municipalities. In 2000, 23% of municipalities had IMR greater than 40 per 1000 LB, mostly in the North and Northeast. In the year 2015, only 2% had an IMR greater than 40 per 1000 LB. The spatial distribution of their IMR showed a much higher homogeneity than in previous years.

Table 3 shows the infant mortality rates in the years 2000 and 2010 by income deciles. For the year 2000, IMR values ranged from 47.9 to 17.3 per 1000 LB, from the first to the last income decile, while in 2010, they ranged from 26.4 to 12.5, respectively. All measures of IMR inequality per income decile declined considerably over the two census years. It is noteworthy that the slope of inequalities, which measures the gradient of decline per income decile, decreases sharply from 2000 to 2010, narrowing to less than half after the 10-year period.

**Table 3:** Infant mortality rate (/1000 live births) by income deciles and summary measures of health inequalities among Brazilian municipalities (Brazil, 2000 and 2010)

Income deciles	IMR	
	2000	2010
1	47.9	26.4
2	43.3	23.2
3	40.5	21.9
4	37.4	21.8
5	30.1	19.8
6	26.2	17.8
7	23.6	14.9
8	21.0	13.9
9	20.2	13.0
10	17.3	12.5
**Summary measures**		
Difference	30.6	13.9
Ratio	2.8	2.1
Slope Index of Inequality	− 3.518	− 1.573

## Discussion

There has been substantial improvement in vital information systems both from the point of view of registration completeness and data quality, when it comes to access to health care and the prioritization of strategies to reach the municipalities with the lowest socioeconomic status [14].

The possibility of using continuous registration data for the elaboration of infant mortality rates in all Federative Units (FU) and Brazilian municipalities from the year 2000 onwards [16, 17] made it possible to estimate the IMR in different geographic cut-offs and to analyze the space–time inequalities.

Mortality in the first year of life, an indicator recognized by its sensitivity to living and health conditions, showed an important decrease between 1990 and 2015. The decrease also pointed out in previous studies [23, 24] undoubtedly reflects the progress achieved in terms of expanding access to medical care [25]. In this period, estimates calculated by the GBD and by the MoH showed the highest annual decreasing rate in the Northeast Region, contributing to the reduction of regional inequalities in infant mortality, which lasted for several decades [10]. The highest IMR reduction in the poorest Brazilian region undoubtedly reflects the benefits related to the expansion of primary health care, which ensured access to basic health services that are important for the health of children and women before, during, and after pregnancy [6, 26, 27].

The Global Burden of Disease (GBD) 2017 study, which included estimates for the Brazilian Federal Units, represents an opportunity for studies aimed at analyzing the country’s geographical inequalities, using a standardized methodology to correct problems of quality and incomplete information. Regarding the magnitude of the rates, however, the IMR GBD estimates were 20% to 30% higher than those obtained by the MoH from 1990 to 2015 and much higher than those reported by the World Bank, calculated by the United Nations interagency group, responsible for estimating infant mortality [20]. Particularly, in the year 2015, the estimated IMR by the World Bank for Brazil was 14.0 per 1000 LB, very close to that estimated by the MoH (13.5), while the GBD estimate was 16.4 per 1000 LB.

Despite the possible IMR overestimation by the GBD, when the estimates of the GBD and the MoH are compared by FU, the correlations are positive and significant, and therefore, similar ratios were obtained between the FU estimates calculated by the two methods. However, in 2015, the IMR estimated by the GBD was 40% higher in the South Region and 50% higher in the states of Paraná and Santa Catarina. This region has the best socioeconomic status in the country and is considered to have the most complete vital information. In the three states of the region, infant mortality rates are calculated directly by their definition without any correction [19]. The largest discrepancy in the South Region indicates the need to review the GBD methodology applied to Brazil at subnational levels.

The results presented in this study undoubtedly show the reduction of inequalities in infant mortality, regardless of the geographic scale and the calculation methodology. At the level of Federative Units, the ratio between the highest and lowest estimates of the IMR was reduced from 4 to 2 between 1990 and 2015, both when using the estimates produced by the MoH and by the GBD. In addition, the spatial distribution of infant mortality in 2015 in Brazilian municipalities outlines a much more homogeneous picture than in previous years.

In Brazil, the focus on inequalities at the regional level has proved especially important to promote actions and programs to reduce the socioeconomic gap. Prioritization of the poorest municipalities has shown significant impacts on reducing the historic regional gap in infant mortality rates, unnecessary hospitalizations, and under-five mortality rate due to undefined causes and unassisted deaths [28].

As a consequence of the considerable decline in infant mortality, the magnitude of the national rate in 2015 was similar to that observed in countries with the same per capita income, something that did not occur until the mid-2000s [2], when the IMR in Brazil was higher than expected according to the World Bank model that relates infant mortality to the per capita GDP of the world’s countries [20]. Northeastern states, however, performed better than those in the Southeast Region, probably due to the lower rates of decrease among neonatal deaths. These findings corroborate those found in the GBD study. Based on the GBD sociodemographic index, estimates of infant mortality rates in some Northeastern states were lower than expected [25].

The comparison of IMR municipal data by income deciles also showed a significant reduction in all measures of health inequality from 2000 to 2010. The decrease in all health inequality measures that go beyond the reduction of income inequality is likely to be reflecting the effects of the income transfer program “Bolsa Família”, which, coupled with the expansion of primary care, has reinforced this effect on child health [29].

While progress in reducing child mortality is evident, there are still challenges to overcome. The current pattern of mortality in the first year of life, which is concentrated in the early neonatal period and presents a lower rate of decrease the closer to delivery, shows the importance of factors related to pregnancy, delivery, and postpartum, generally preventable through quality health care [30, 31].

Despite the marked reduction of inequalities in various indicators of maternal and child health and the remarkable expansion of coverage of prenatal care and hospital delivery after the implementation of the Unified Health System [32], additional efforts aimed at improving access to good quality childbirth care are crucial. It is necessary to integrate actions developed in primary care to the services of childbirth care, to potentiate the municipal capacities in providing adequate assistance to maternal and delivery care [33].

In this context, we highlight the importance of monitoring the perinatal mortality rate, considered as a key health outcome for interpreting the impact of maternal–child health actions [34, 35]. In Brazil, as in other countries, the analysis of perinatal mortality statistics faces additional difficulties, since definitions of stillbirth are not always obeyed [36], and thousands of newborns are not registered as having been born [37]. In 2015, the reported number of stillbirths weighing more than 2500 g was higher than 8000, with almost 70% of those carried for 37 or more weeks of gestation, indicating that many of the deaths occurring shortly after delivery may have been misclassified as fetal deaths.

Other difficulties faced are the recent cuts in social investments as well as in the Unified Health System [38]. The recent economic crisis led to a reduction in private health plan users, and, consequently, to an increase in demand for public services [10]. Despite the progressive decrease in IMR between 2010 and 2015, analyses of infant mortality in 2016 showed a 3% increase in relation to the 2015 estimate, which has raised concerns on the IMR trends after budgetary constraints.

The increase in IMR in 2016 was not only due to the decrease in the number of live births in 2016, probably attributed to the proportion of women who avoided pregnancy soon after the Zika virus epidemic, but also to the increase in the number of infant deaths in the post-neonatal period, in the number of infant deaths due to diarrhea, and in the proportion of ill-defined deaths, while deaths due to congenital anomalies continued to decrease. The increases, although punctual, occurred for specific categories, which were all associated to the worsening of living conditions and lack of access to medical care. Although post-2016 mortality data are not available to assert that there is a reversal in IMT trends, the increases in specific causes seem to reflect the cuts in social policies in recent years.

The limitations of this study refer to the estimates of infant mortality. Although underreporting of deaths and live births has decreased considerably throughout the country, it is not yet possible to estimate the IMR directly, by the ratio of the number of child deaths per 1000 live births. Given the restrictions on the use of mortality estimates based on demographic methods [12], efforts have been made in Brazil to improve the vital information systems and use more appropriate correction factors based on empirical results of active search studies of deaths and births conducted in 2010 and 2014. However, the factors used to adjust the number of deaths and live births may not be adequate for some municipalities. Another limitation is the probable misclassification of newborns who die shortly after birth as fetal deaths, underestimating the infant mortality rate.

## Conclusion

The progress achieved in reducing child mortality after the implementation of the Unified Health System and the expansion of primary health care is undeniable, not only in terms of the magnitude of the IMR, but also in terms of the reduction of geographical and income inequalities. The comparison of the IMR GBD estimates with the national ones shows ever greater magnitudes for the GBD rates. However, despite the possible IMR overestimation by the GBD, the FU annual decrease rates in the period 1990–2015 are similar.

## Data Availability

Data we used in this article are publicly available online on the official website of Institute of Health Metrics and Evaluation (http://ghdx.healthdata.org/gbd-results-tool) and on the official website of the Ministry of Health (http://datasus.saude.gov.br).

## Abbreviations

FU: Federative Units
GBD: Global Burden of Disease
GDP: Gross Domestic Product
GMR: Age-standardized general mortality rate
HIV: Human immunodeficiency virus
IMR: Infant mortality rate
LB: Live births
MoH: Ministry of Health
SIM: Mortality Information System
SINASC: Live Birth Information System
VR: Vital Registry
